# The effect of platelet-rich plasma on the improvement of pregnancy results in repeated implantation failure: A randomized controlled trial

**DOI:** 10.18502/ijrm.v20i9.12065

**Published:** 2022-10-10

**Authors:** Elaheh Baybordi, Jafar Mohseni, Parisa Mosapour

**Affiliations:** ACECR Infertility Treatment Center, Tabriz, Iran.

**Keywords:** Platelet-rich plasma, Pregnancy results, Embryo implantation.

## Abstract

**Background:**

The success rate of infertility treatment depends on many different factors.

**Objective:**

This study aimed to determine the effect of platelet-rich plasma (PRP) on the improvement of pregnancy outcomes in participants with repeated implantation failure.

**Materials and Methods:**

The study is a randomized triple-blind clinical trial. The study population was 118 women with repeated implantation failure during assisted reproductive technology treatment at Tabriz Jihad-e Daneshgahi ART Center from May 2017 to December 2019. Intervention: Intrauterine injection of autologous PRP. Standard treatment of fetal transfer to the uterine cavity was performed without intrauterine PRP injection in the control group: After 4 wk, the level of β-human chorionic gonadotropin hormone in participants' blood was measured.

**Results:**

Comparing the effect of intrauterine injection of PRP in 2 groups showed the level of β-human chorionic gonadotropin positive in the intervention group was 21 (43.8%), in the control group was 12 (26.1%), odds ratio = 2.20 (0.92-5.26) and p = 0.073.

**Conclusion:**

The therapeutic effect in the intervention group compared to the control regarding the outcome of a successful pregnancy showed that intrauterine injection of PRP can be effective in improving pregnancy outcomes, although this improvement is not significant.

## 1. Introduction 

The success rate of infertility treatment depends on many different factors, the most important of which are maternal age, causes of infertility, the weak response of the ovaries to stimulation, the influence of male factors, sperm quality, fetal quality, and various uterine pathologies. Some couples experience repeated implantation failure (RIF) after embryo transfer (ET) despite acceptable quality and without any major problems (1). According to the European Society of Human Reproduction and Embryology consortium, PIF is known as absence of a gestational sac on ultrasound at 5 wk or following 3 ET with good quality embryos or after the transfer of 10 or more embryos in multiple transfers (2).

As yet, several methods have been proposed for management of RIF. Assisted hatching, pre-implantation genetic screening, blastocyst transfer, co-culture system, hysteroscopy, sequential transfer, endometrial scratching, extra number ET, natural cycle, salpingectomy for tubal disease, oocyte donation, intra tubal ET, immune therapy, and endometrial receptivity array have been used but there has not been any proven evidence of effectiveness in these treatments (3-5).

There have been studies on the effectiveness of platelet-rich plasma (PRP) therapy, and intrauterine injection of PRP has been proposed to stimulate growth and endometrial acceptance. PRP is derived from fresh whole blood taken from a peripheral vein and prepared. It is stored in a solution of citrate acid dextrose A and anticoagulant and is processed to increase platelets by isolating various components of the blood. During platelet activation in PRP, cytokines and growth factors are activated and released in 10 minutes after clotting. These factors include transforming growth factor, platelet-derived growth factor, vascular endothelial growth factor, and epidermal growth factor. These substances cause cell migration, binding, proliferation, differentiation, and stimulation of extracellular matrix accumulation (6). The effectiveness of intrauterine PRP injection is not known in participants who have had unsuccessful implantations for some reason, and studies have been carried out on small sample sizes and no comparison has been made.

This study aimed to determine the effect of PRP on the improvement of pregnancy outcomes in participants with RIF to examine the potential positive effects of the new treatment method to increase the success rate of pregnancy in participants undergoing assisted reproductive technology (ART) treatments.

## 2. Materials and Methods

The study is a randomized triple-blind clinical trial. The inclusion criteria for participants in the study were women of childbearing age with a history of RIF during ART treatments. Exclusion criteria were women aged > 18 and aged < 45 yr old, diagnosed cancers, anemia as known hemoglobin less than 11 gr per deciliter, platelets count less than 150,000/ cc, pregnancy, use if anticoagulants, use of non-steroidal anti-inflammatory drugs up to 10 days before the procedure, any physical or mental illness that affects the participant's immunity and admission and disrupt the process of implantation and participants follow-up.

This study was performed at Tabriz Jihad-e Daneshgahi ART Center, Tabriz, Iran between May 2017 to December 2019. Study population was participants with RIF during ART treatment. The intervention was intrauterine injection of autologous PRP.

All participants underwent standard assisted reproduction therapy after obtaining written informed consent, interviewing the participant and explaining the plan. Endometrial thickness was measured by transvaginal ultrasound, and if the thickness was 8 mm and the fetus was acceptable, intrauterine PRP autologous injection was performed on participants in the intervention group.

PRP was taken from the participant's autologous blood simultaneously, then prepared by centrifugal platelets and growth factors isolated and concentrated from the participant's serum, after that 0.5-1 ml of PRP was injected into the uterine cavity on day 10 ${}^{\text{th}}$ of the HRT cycle (hormone replacement therapy) and repeated as needed. After 48 hr of PRP injection, which was performed once or twice per cycle, the embryos were transferred into the uterine cavity in the blastocyst stage based on the standard protocol. Standard treatment of fetal transfer to the uterine cavity was performed without intrauterine PRP injection in the control group.

### Outcomes

After 4 wk, the level of β-human chorionic gonadotropin (β-HCG) hormone in participants' blood was measured and the presence of a pregnancy sac was confirmed in both intervention and control groups. The participant was followed up after 3 wk to check the fetus heart rate and monitor the pregnancy process. Then, if the pregnancy proceeded successfully until the 40 wk of pregnancy, the participants were placed under standard care, and finally live births, abortion and ectopic pregnancy were reported.

### Sample size

Using the G POWER software and the following formula to determine the sample size in 2 independent groups and to study non inferiority and superiority with β = 80%, α = 0.05 and EF = 60% (based on published articles), a sample size of 94 people, 48 people in intervention group and 46 people in control group, were identified, and 5 more samples were taken to prevent attrition rate.

Randomization and allocation concealment methods included: 118 participants referred to Tabriz Jihad-e Daneshgahi ART Center with RIF during ART treatment and signed informed consent to participate in the study. The participants were divided into 2 groups of intervention and control using the sealed envelopes randomization method participants selected one of the envelopes and delivered it to the relevant observer who was unaware of the meaning of the codes. The letters A and B were written inside the envelope and the participants were divided into 2 groups so that each participant had an equal chance of being assigned to each group.

Sham surgery method was used for the intervention group. PRP kits were provided for all participants and they were unaware of whether or not intervention would be performed in the operating room. The outcome variable was reported by the sonographer, and the participant and physician were still unaware of the participants' group allocation. The final analyst was also unaware of the group allocation. PRP kit was prepared for all participants and each participant was unaware of the PRP use in the course of ET throughout the intervention.

Clinical trials were conducted by individuals with relevant qualifications from the scientific point of view including gynecologists with infertility fellowship, geneticists, embryologists, and social medicine specialists. PRP therapy has had no side effects so far, and previous studies have shown the benefits of this treatment. All necessary precautionary measures were taken to protect the privacy of the subjects, as well as reduce the adverse effects of the study on their physical and mental health. Necessary arrangements have been made for them to access to the best methods of prevention, diagnosis, treatment, or other appropriate care.

### Ethical considerations

This study has been approved by the ethics committee of Ethics Committee of Royan Research Institute of Jihad-e Daneshgahi, Tehran, Iran (Code: IR.ACECR.ROYAN.REC.1396.183). This trial was registered on 10.7.2017 on the website www.irct.ir.

### Statistical analysis

The analyst blindly analyzed each group with code and made comparisons between the 2 independent groups. The SPSS (ststistical pakage for social sience) software version 16 IBM Company reported the results of the project using the Kolmogorov-Smirnov, Chi-square statistical tests, Fisher's exact test, descriptive evidence, and a 95% confidence interval. P-value < 0.05 was considered significant.

## 3. Results 

**Figure 1:**
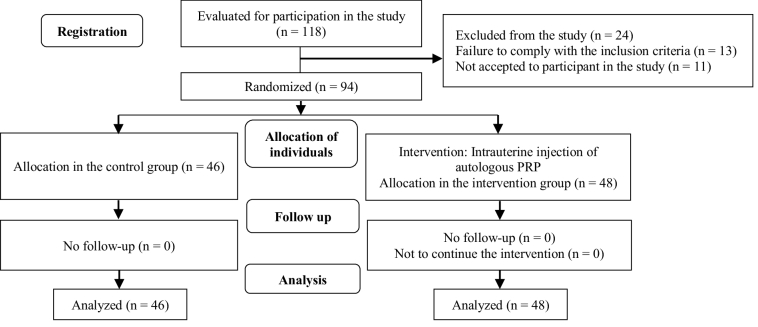
Consolidated standards of reporting trials (CONSORT) flowchart.

The process flow chart based on CONSORT statement is shown in figure 1. The follow-up period of each participant was based on the outcome of the intervention or non-intervention process. Participants who were monitored with or without intrauterine PRP after ET underwent blood tests and blood levels of β-HCG 4 wk later and if the results were positive, they underwent an ultrasound 3 wk later to examine the sac and fetus's heart. Specialized follow-ups and visits were performed in case the fetus' heart rate was positive until the end of the pregnancy. The ongoing intrauterine pregnancy diagnosis, ectopic, abortion, live birth were checked by ultrasound during the pregnancy. Participants whose test and ultrasound results were negative were not followed up with. The study was completed in February 2018 and lasted 21 months. The minimum follow-up time for participants was 2 months and the maximum was 10 months. The study was completed after following up and obtaining the results of all participants. The study population was 94 women, of whom 48 were in the intervention group and 46 in the control group at random. The basic information of the participants is shown in table I.

Analysis of the final results of the intervention group and the effect of intrauterine injection of platelet-rich autologous plasma on the success of implantation have been reported in table II.

Relative risk reduction or in other words, the progress made in obtaining a positive result from the intervention on the laboratory outcome of HCG positivity was 68% in both groups. In terms of the consequences of recognizing a fetus with ultrasound in the 5 ${}^{\text{th}}$ and 7 ${}^{\text{th}}$ wk, the relative risk reduction was 52% and 36%, respectively. The rate of number needed to treat or the number of treatments required for this intervention was calculated to be 10.75.

Relative improvement was obtained in the outcome of the pregnancy. Live births and pregnancies over 20 wk between groups was 64%, and the number needed to treat was calculated to be 7.25. Due to the fact that no participants in both groups were excluded from the study, the analysis of intention to treat and per-protocol had the same result. In terms of the risks caused by the study, it should be noted that since the content of the injected material in the intervention group included participants' PRP and the control group receiving the standard treatment, no side effects or problems affected the participants and all intervention and control processes were performed under sterile operating room conditions to avoid the risk of infection transmission.

**Table 1:** Basic characteristics of the participants studied in each group


**Variable **		**Intervention group (n = 48)**	**Control group (n = 46)**
Age*		37.33 ± 6.439	32.41 ± 5.651
Body mass index*		26.64 ± 3.302	26.86 ± 3.63
Infertility duration (yr)*		12 ± 6.16	7 ± 4.71
Number of embryos transferred*		2.46 ± 0.74	2.52 ± 0.75
Age of the embryos (days)*		3.71 ± 0.85	3.67 ± 0.82
Infertility type**			
	**Primary**	36 (75)	39 (84.8)
	**Secondary**	12 (25)	7 (15.2)
Fertility history**			
	**Live child**	2 (4.2)	3 (6.5)
	**Dead child**	2 (4.2)	1 (2.2)
	**Miscarriage**	13 (27)	8 (17.3)
	**Ectopic pregnancy**	2 (4.2)	6 (13)
Medical history**			
	**Diabetes**	1 (2.1)	1 (2.2)
	**Hypertension**	1 (2.1)	0 (0)
	**Poly cystic ovarian syndrome**	1 (2.1)	0 (0)
	**Hyper hypothyroidism**	0 (0)	10 (21.7)
	**Hyper prolactinemia**	2 (4.1)	1 (2.2)
	**Anxiety disorder**	1 (2.1)	0 (0)
	**Clinical depression**	0 (0)	3 (6.5)
Drug history**			
	**IVIG**	7 (14.6)	11 (23.9)
	**Subcutaneous enoxaparin**	41 (85.4)	30 (65.2)
	**Aspirin**	29 (60.4)	36 (78.3)
	**Levothyroxine**	0 (0)	13 (28.3)
	**Metformin**	2 (4.1)	7 (15.2)
*Data presented as Mean ± SD. **Data presented as n (%). IVIG: Intravenous immune globulin			

**Table 2:** Comparing the effect of intrauterine injection of platelet-rich autologous plasma in intervention and control groups


**Variable**	**Intervention group**	**Control group**	**Odds ratio (95% CI)**	**P-value**
**Chemical pregnancy**	21 (43.8)	12 (26.1)	2.20 (0.92-5.26)	0.073
**Clinical pregnancy (4 wk) **	19 (39.6)	12 (26.1)	1.86 (0.77-4.46)	0.164
**Clinical pregnancy (7 wk)**	17 (35.4)	12 (26.1)	1.55 (0.64-3.56)	0.32
**Live birth**	9 (18.8)	8 (17.4)	1.09 (0.38-3.13)	0.864
**Ongoing pregnancy**	8 (16.7)	2 (4.3)	4.4 (0.88-21.96)	0.053
**Abortion**	2 (4.2)	2 (4.3)	0.96 (0.13-7.09)	0.965
**Ep**	1 (2.1)	0 (0)	1.02 (0.98-1.06)	0.325
Data presented as n (%). Chi-square and Fisher's exact test. P-value < 0.05 is significant. CI: Confidence interval, EP: Ectopic pregnancy				

## 4. Discussion

This study is a superiority trial type and has been conducted to evaluate the therapeutic effect of intrauterine injection of PRP in participants who suffer from RIF undergoing assisted reproduction methods. The results of this study measured the therapeutic effect in the intervention group compared to control regarding the outcome of a successful pregnancy, including live birth and pregnancy over 20 wk, which was 64%. Comparison of the 2 groups in the hormone-positive variable showed that the odds ratio of success of pregnancy outcome in the intervention group compared to the control group was 2.20 (0.92-5.26), which was not significant. Given that the confidence interval shows the relationship between the chance and the p-value, the comparison between the 2 groups to study the effect of intrauterine PRP injection was not statistically significant, which may be due to the small sample size.

In another study the effect of PRP on improving pregnancy rates in RIF participants have been examined, a number of women with a history of RIF was selected to transfer frozen embryos. Intrauterine injection of 0.5 ml of PRP which contained platelets 4-5 times more than the peripheral blood samples was done 48 hr before the transfer of blastocyst, 18 women became pregnant, one premature miscarriage and one molar pregnancy. 16 pregnancies were successful and going on. In this study, PRP appeared to be effective in improving pregnancy outcomes in RIF participants (2). The study of Farimani et al. which was a one-way blind study was conducted for the first time in Hamadan, Iran in 2016. The study was conducted to examine the hypothesis that intrauterine use of PRP could improve the outcome of pregnancy by transferring frozen embryos to infertile women with a history of RIF. Ultrasound replacement therapy was performed on the uterine cavity under ultrasound guidance using a Wallace catheter about 36 hr before the ET and then 0.5-1 ml of PRP was injected. The number of participants was 9, and the clinical pregnancy was confirmed by blood β-HCG measurement 14 days after an ET in 6 of the participants. The average successful pregnancy was reported to be 66.6%. Despite the limitations of a preliminary study, including the small sample size and the lack of a control group, this preliminary study suggests that PRP injection before ET may play a vital role in successful implantation. This study is currently in progress and the final results have not yet been reported (7).

Another study compared the effects of PRP and granulocyte colony stimulating factor on infertile women with a history of RIF, which was a retrospective cohort study. The clinical pregnancy rate in the PRP group was 40.3% vs. 21.4% in the GCSF group with p = 0.025 and showed a positive effect of intrauterine PRP injection (8). In the PRP evaluation study, the transfer of frozen embryos in participants whose endometrial thickness was not accepted and had a history of RIF, a positive and significant effect on increasing endometrial thickness was also recorded, which was effective in implantation and pregnancy (9). A recently published systematic review study examined 7 studies that highlighted the positive role of intrauterine PRP in women with a history of RIF and the transfer of frozen embryos and it has been introduced as a way to increase the possibility of successful pregnancies in assisted reproduction methods (10).

### Limitation

Performing previous treatments and taking medications in previous treatments and satisfying the patient to perform the intervention are the limitations of the study.

## 5. Conclusion

Measuring the therapeutic effect in the intervention group compared to control in the outcome of a successful pregnancy showed that intrauterine injection of PRP can be effective in improving pregnancy outcomes, but this improvement is not significant.

##  Conflict of Interest

The authors declare that there is no conflict of interest.
